# Correlation of EGFR, pEGFR and p16^INK4^ expressions and high risk HPV infection in HIV/AIDS-related squamous cell carcinoma of conjunctiva

**DOI:** 10.1186/1750-9378-9-7

**Published:** 2014-02-26

**Authors:** Anthony Mwololo, Joshua Nyagol, Emily Rogena, Willis Ochuk, Mary Kimani, Noel Onyango, Lorenzo Pacenti, Rosa Santopietro, Lorenzo Leoncini, Walter Mwanda

**Affiliations:** 1 Department of Human Pathology, University of Nairobi, Nairobi, Kenya; 2 Department of Anatomical Pathology and Human Oncology, University of Siena, Siena, Italy; 3 Department of Human Pathology, Unit of Immunology, School of Medicine, College of Health Sciences, University of Nairobi, P.O. Box 19676-00202, KNH (Off Ngong Road), Nairobi, Kenya

**Keywords:** SCCC, Biomarkers, HPV, HIV/AIDS

## Abstract

**Background:**

Squamous cell carcinoma of conjunctiva has increased tenfold in the era of HIV/AIDS. The disease pattern has also changed in Africa, affecting young persons, with peak age-specific incidence of 30-39 years, similar to that of Kaposi sarcoma, a well known HIV/AIDS defining neoplasm. In addition, the disease has assumed more aggressive clinical course. The contributing role of exposure to high risk HPV in the development of SCCC is still emerging.

**Objective:**

The present study aimed to investigate if immunohistochemical expressions of EGFR, pEGFR and p16, could predict infection with high risk HPV in HIV-related SCCC.

**Methods:**

FFPE tissue blocks of fifty-eight cases diagnosed on hematoxylin and eosin with SCCC between 2005-2011, and subsequently confirmed from medical records to be HIV positive at the department of human pathology, UoN/KNH, were used for the study. Immunohistochemistry was performed to assess the expressions of p16INK4A, EGFR and pEGFR. This was followed with semi-nested PCR based detection and sequencing of HPV genotypes. The sequences were compared with the GenBank database, and data analyzed for significant statistical correlations using SPSS 16.0. Ethical approval to conduct the study was obtained from KNH-ERC.

**Results:**

Out of the fifty-eight cases of SCCC analyzed, twenty-nine (50%) had well differentiated (grade 1), twenty one (36.2%) moderately differentiated (grade 2) while eight (13.8%) had poorly differentiated (grade 3) tumours. Immunohistochemistry assay was done in all the fifty eight studied cases, of which thirty nine cases (67.2%) were positive for p16INK4A staining, forty eight cases (82.8%) for EGFR and fifty one cases (87.9%) showed positivity for p-EGFR. HPV DNA was detected in 4 out of 40 SCCC cases (10%) in which PCR was performed, with HPV16 being the only HPV sub-type detected. Significant statistical association was found between HPV detection and p16INK4 (p=0.000, at 99% C.I) and EGFR (p=0.028, at 95% C.I) expressions, but not pEGFR. In addition, the expressions of these biomarkers did not show any significant association with tumor grades.

**Conclusion:**

This study points to an association of high risk HPV with over expressions of p16INK4A and EGFR proteins in AIDS-associated SCCC.

## Background

Squamous cell carcinoma of conjunctiva (SCCC) is a rare, slow-growing tumor of a spectrum of conditions collectively known as ‘ocular surface epithelial dysplasias’. These range from benign dysplasias, to carcinoma *in situ* and ultimately to invasive carcinoma [1,2]. It is also considered the most common neoplasm of the conjunctiva, normally affecting elderly men of around 70 years [3,4]. The etiology of SCCC has been previously viewed as multifactorial, with ultraviolet light implicated as the major risk factor for these tumors, and more common in equatorial Africa than in Europe or North America [5].

In Africa, however, the incidence of SCCC has risen rapidly in the recent past, with the peak age-specific incidence reported to be 30-39 years, similar to that of HIV/AIDS pandemics in the tropics. The disease is also more aggressive, with a mean history of three months at presentation [4,5]. An association between human immunodeficiency virus (HIV) infection and squamous cell carcinoma of the conjunctiva was first reported in the mid-1990s. Subsequently, other studies also reported that since the advent of HIV/AIDS in 1980s, the number of patients presenting with SCCC had been increasing exponentially [6-8]. In 1995, Ateenyi-Agaba observed that a high incidence of these tumors in Uganda appeared to be related to HIV infection [9]. Parallel studies by Waddell and colleagues also suggested that HIV infection is strongly associated with an increase in the incidence of conjunctival carcinoma in Africa [10].

Although the natural history of the SCCC appears to be unique in this region of the world with etiologic mechanism unclear and therapeutic options limited, immunosuppression from HIV has been thought to facilitate the activity of other infective agents that induce the carcinoma. This has been supported by many studies that have documented the presence of high risk HPV genotypes 16 and 18 DNA in a proportion of SCCC [11]. The oncoproteins E6 and E7 encoded by the high risk HPV genotypes are well documented to play a critical role in pathogenesis of anogenital carcinomas by deregulation of cell cycle control proteins p53 and pRb2/p130, respectively [12-18].

Another cell cycle regulatory protein whose increased expression is reported to be predictive of, and an independent prognostic marker in high risk HPV infection is p16^INK4A^[19-22]. However, in our previous study to investigate the roles played by HIV-1 Tat and high risk HPV E6/E7 proteins in promoting carcinogenesis in cervical cancers, expressions of p16^INK4A^ was found to be reduced in HIV-related squamous cell carcinoma of the uterine cervix, a correlation which has also been supported by other studies [23,24].

Few authors have also proposed involvement of high risk HPVs in the oncogenesis through altered expression of other key molecules involved in tyrosine kinase pathways, in which inverse expressions of epidermal growth factor receptor (EGFR) and its phosphorylated form (pEGFR) have been reported to contribute to the pathogenesis of HIV/AIDS–associated SCCC, and correlates with poor prognosis [25-27]. Altogether, expressions of p16^INKA4^, EGFR and its phosphorylated form pEGFR have been proposed to reflect infection with high risk HPV in squamous cell carcinoma of conjunctiva. However, HPV detection in conjunctival neoplasm has been largely controversial, and the pathogenic mechanism not well elucidated. Some of these studies have reported a heterogeneous prevalence of high-risk HPV genotypes, suggesting that only a subset of cases can be attributed to these viruses. This variation in the reported HPV infection rates in conjunctival squamous cell carcinoma could result from differences in detection methods and the studied populations [28-31].

The present study therefore compared PCR-based detection of HPV with immunohistochemical expressions of p16^INK4A^, EGFR and pEGFR; and the potential value of use of these biomarkers as indicators of HPV positivity in selected cases of HIV/AIDS-related SCCC.

## Results

### Patient’s demographics and histology

**Figure 1:**
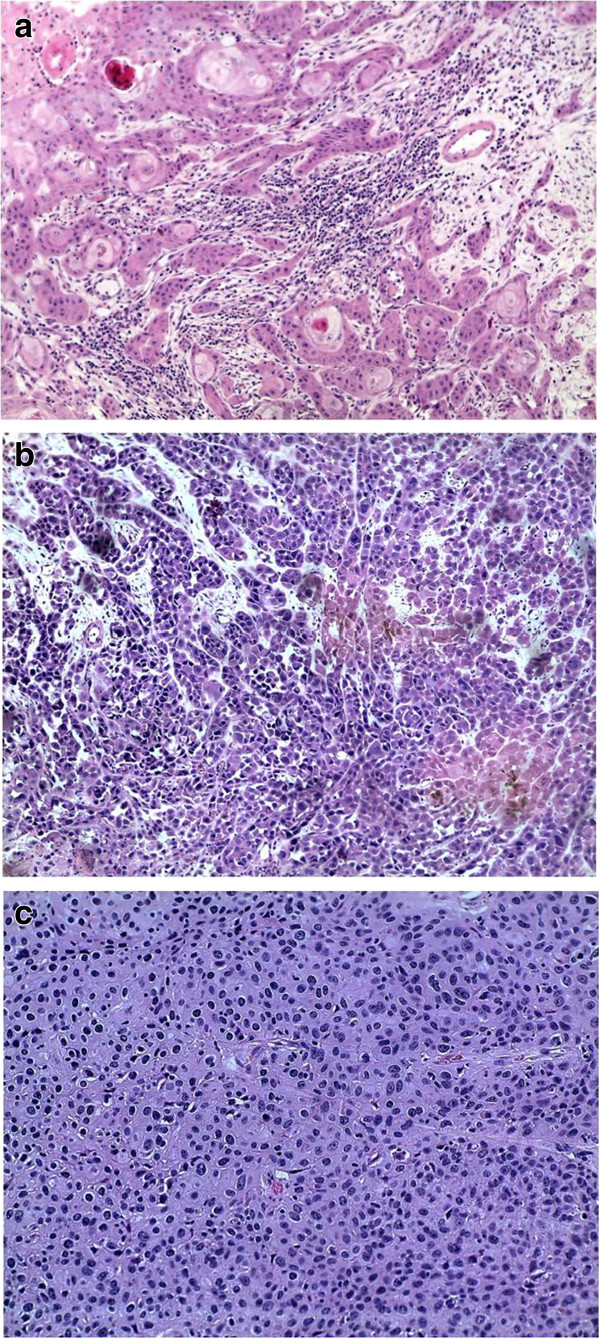
**Haematoxylin and eosin staining. a**. Grade I, SCCC X 10 Neoplasm comprising of infiltrating cords of mild to moderately pleomorphic malignant squamous cells and of keratin pearls, interspersed with a chronic inflammatory cell infiltrate). **b**. Grade II SCCC X 10 (Neoplasm comprising of infiltrating cords of moderately pleomorphic malignant squamous cells). **c**. Grade III SCCC X 20 (Neoplasm disposed in a diffuse architecture, comprising of moderately to markedly pleomorphic malignant squamous cells).

Out of the 58 samples evaluated, the ratio of females to males was 1:1. The age ranged from 23 to 73 years, with a mean of 41.6 years and median of 39 years. Fifty six cases were classified as squamous cell carcinomas and the remaining two as carcinoma *in situ*. Tumor site, tumor size and tumor grades were as shown in Table 1 and Figure 1a-c.

**Table 1:** n = 58

	**Total**	**%**
*Age*		
<50 years	9	15.5
>50 years	42	72.4
Unknown	7	12.1
*Sex*		
Male	29	50%
Female	29	50%
*Tumor site*		
Right orbit/eye/conjunctiva	22	37.9
Left orbit/eye/conjunctiva	24	41.4
Orbit/eye/conjunctiva	12	20.7
*Tumor size*		
<2 cm	36	62.1
2-5 cm	6	10.3
>5 cm	15	25.9
Unknown	1	1.7
*Tumor grade*		
I	29	50
II	21	36.2
III	8	13.8

### Expressions of p16^INK4A^, EGFR and pEGFR

**Figure 2:**
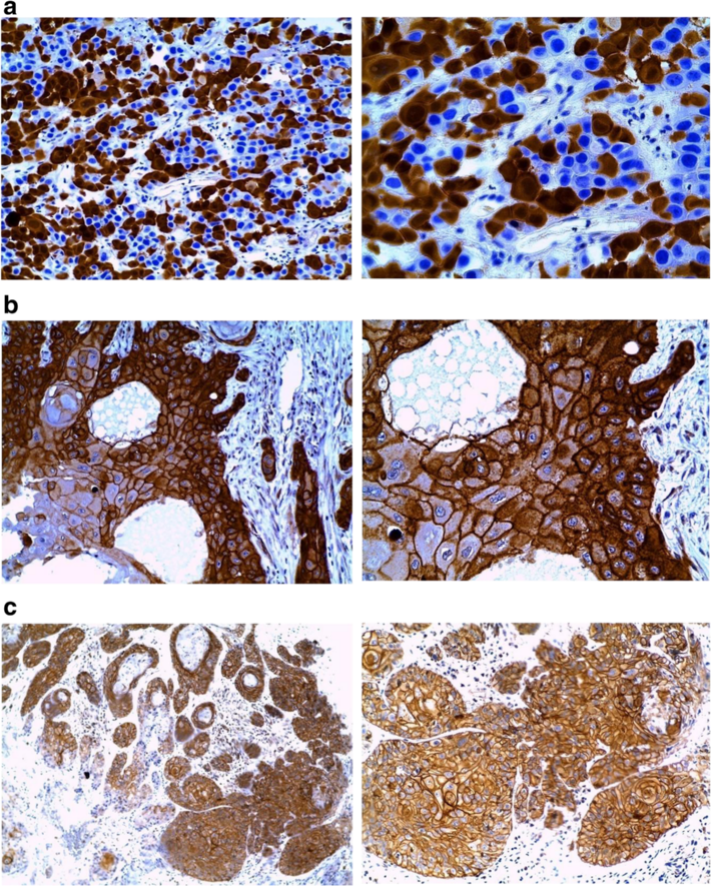
**a1 (X20) and a2 (X40).** Staining for P16^INK4A^ in majority of the neoplastic cells showed intense nuclear staining, which reflects the functional role of p16 in control of cell cycle prior to the S-phase. In some cells, cytoplasmic positivity appeared prominent. **b1** (X20) and **b2** (X40). EGFR staining was observed to be brown membrane and cytoplasmic, uniformly distributed in all the squamous epithelial cells. **c1** (X10) and **c2** (X20). Staining for pEGFR showed complete brown membrane and cytoplasmic reaction in all epithelial tumors cells.

Immunohistochemistry assay was done in all the fifty eight studied cases, of which Out of the fifty eight cases, thirty nine (67.2%) were positive for p16^INK4A^ staining, forty eight cases (82.8%) for EGFR and fifty one cases (87.9%) showed positivity for p-EGFR (Figure 2a-c).

The patterns of p16^INK4A^, EGFR and pEGFR expressions were semi-quantitatively scored for both intensity and proportion of staining in the cell nucleus, cytoplasm and membrane as reported previously [32,33]. Intensity of the staining were scored as 0 (no staining), 1 (weak positivity), 2 (moderate positivity), or 3 (strong positivity). The proportions of the staining were also evaluated and scored as 0 (1%-10% of cells stained), 1 (11%-50%), 2 (51%-80%) or 3 (81%-100%). Sections scored as 0 or 1 for intensity was defined as negative; whereas those scored 2 or 3 were defined as positive.

### HPV 16 is the predominant genotype in AIDS- related SCCC

From the forty cases where DNA was extracted, four cases (10%) showed positivity for HPV 16 genotype, and no other genotypes were detected. Although this is in tandem with other studies that have documented detection ranges from 8-10% of high risk HPV in head and neck squamous cell carcinomas, this low rate of detection suggests that other mechanisms could underlie pathogenesis of HIV/AIDS-related squamous cell carcinoma of conjunctiva independently from the active role of high risk HPV.

### Correlations of HPV detection with p16^INK4A^, EGFR expressions

For the cases in which high risk HPV were positive, statistical significant association was found between HPV detection and p16^INK4^ expressions, p = 0.000, at 99% confidence interval and EGFR expression, p = 0.028, at 95% C.I, but not with pEGFR.

## Discussion

A causal relationship between the epidemic of HIV/AIDS in the Sub-Saharan region and increased incidence of squamous cell carcinoma of conjunctiva (SCCC) has been hypothesized, with the prevalence of the neoplasm reported in Kenya alone to be 8% [34,35]. Although immune suppression has been known to play a critical role in the pathogenesis, a number of epidemiological studies have postulated other etiological agents for the development of squamous cell carcinoma of conjunctiva, such as ultraviolet (UV) light, history of pterygium, and high risk human papillomavirus infection (HPV) [36-39].

Detection of high risk HPV associated with squamous cell carcinoma has, however, been controversial, with different authors reporting varied percentages of positivity and prevalence [40-42]. This could be attributed to various techniques currently in use, ranging from consensus and type-specific end-point PCR methods followed with sequencing, real-time PCR assays for quantification of viral load, *in-situ* hybridization, detection of serum antibodies directed against HPV epitopes, to immunohistochemical detection of surrogate biomarkers such as p16^INK4A^, a cyclin dependent kinase inhibitor protein [42-45]. In our study, a prevalence of 10% of high risk HPV 16 genotype detection was realized, which is consistent with the findings of other studies. Many infectious oncogenic viruses yet to be discovered can use the same mechanism as of high risk HPV in a state of immunosuppression, but the low prevalence in our study could also be a factor attributed to poor tissue processing and thus DNA degradation in a number of the studied cases.

A number of studies have therefore demonstrated that p16^INK4A^ protein over-expression may serve as a surrogate biomarker for biologically and clinically relevant high risk HPV infection in squamous cell carcinoma of the conjunctiva, as well as squamous cell carcinoma and glandular epithelial dysplasia of the uterine cervix [44,45]. Besides, over expression of p16^INK4A^ has also been shown to correlate with the degree and behavioral characteristics of both cervical as well as head and neck neoplasm [46,47].

In addition to over expression of p16^INK4A^ as potential surrogate marker of HPV-derived squamous cell carcinomas, the development of SCCC in HIV positive patients has been documented to involve activation of epidermal growth factor receptor (EGFR) and its phosphorylated form (p-EGFR) [48-50]. The relationship between HPV status and levels of EGFR protein expression with clinical outcome has been reported in several studies, indicating that the best outcomes are observed in patients with HPV-positive tumors with low EGFR expression [50-52]. However, an inverse relationship between HPV status and EGFR gene amplification has also been reported.

Several studies have discussed about the optimal method for determining HPV status on FFPE tumor sections (5, 34, 35), and in the current study, we evaluated immunohistochemical expressions of p16^INKA^, EGFR and p-EGFR IHC as markers of HPV status and utilized HPV PCR in a subset of cases to correlate the results. Immunohistochemistry was performed in all the fifty eight studied cases, and semi-quantitative scores of the bio-markers enumerated. However, 18 cases were excluded from HPV detection by PCR technique, followed with sequencing, because tumor tissue was either inadequate or had extensive DNA degradation due to poor tissue processing. A prevalence of 10% of high risk HPV 16 genotype detection was realized, which is consistent with the findings of other studies. However, the low prevalence of HPV could be associated with poor tissue processing.

In this study p16^INK4A^ expression in the epithelium was characterized by variable, weak to strong, diffuse nuclear and cytoplasmic staining. Thirty nine cases (67.2%) were positive for p16^INK4A^ staining and showed significant statistical correlation between HPV positivity and p16^INK4A^ expression (correlation coefficient -0.502, *p* = 0.000, at 99% confidence interval (C.I)). This was in agreement with similar findings from other authors who demonstrated that p16^INK4A^ expression was significantly associated with the presence of HPV-16 [20].

The expression of EGFR and its activated form, p-EGFR, were also independently enumerated in all of the fifty-eight cases, from which the results demonstrated that 82.8% of the cases expressed EGFR while 87.9% expressed p-EGFR as brown membranous and/or cytoplasmic immunostaining, similar to the findings from other studies [53].

Once EGFR is activated into p-EGFR, it undergoes internalization, resulting in a marked decrease in the non-activated membrane-bound EGFR [54]. For correlation with HPV positive cases, significant statistical correlations was obtained with EGFR expression (correlation coefficient -.252, p = .028 at 95% confidence interval). There was, however, no statistical significant correlation between HPV status and p-EGFR at both 95% and 99% confidence Intervals. Equally, no biomarker showed significant statistical correlation with tumor grades.

Therefore, both immunohistochemical assay and PCR-based techniques showed close agreement in the use of p16^INK4A^ and EGFR but not p-EGFR as clinically useful surrogate biomarkers for high risk HPV infection in HIV/AIDS-associated squamous cell carcinoma of conjunctiva.

## Conclusion

Although the exact role played by high risk human papillomavirus in carcinogenesis is not well known, to the best of our knowledge, this is the first study to show an association of high risk HPV infection with p16^INK4A^ and EGFR over expressions in HIV/AIDS-associated squamous cell carcinoma of conjunctiva.

## Materials and methods

### Selection of the study cases

Fifty-eight HIV positive SCCC formalin fixed paraffin embedded (FFPE) tissue blocks were retrieved from the archives at pathology department, UON/KNH for analysis. The criteria for inclusion as HIV positive case included information from the clinician in the patient’s histological request form indicating on follow-up at the comprehensive care centre, on HAART treatment, ARV treatment, immunnosuppression, p24 marker reactive, retrovirus disease and HIV positive. Sections (4 μm) from the tissue blocks were stained with *hematoxylin* and *eosin* to confirm the previous diagnoses and establish the tumor grades according to the WHO guidelines. Ethical approval to conduct the study was obtained from KNH-ERC.

### Immunohistochemistry tests for p16^INK4A^, EGFR and pEGFR

Immunohistochemistry was performed as described previously by Russo G, *et al*. [55]. Briefly, 4 μm sections of the FFPE tissue blocks were mounted on positively charged slides and incubated overnight incubation at 65 º C. Sections were subsequently de-waxed through a series of xylene, rehydrated in graded series of alcohol (absolute, 90% and 75%), and taken to water. Antigen retrieval was done by immersing the slides in Tris buffer pH 9 containing 10 mmol/l EDTA and 15 mmol/l sodium azide (NaN_3_) (for *p16*^*INK4A*^) or citrate buffer, pH 6.0 (for EGFR and pEGFR) and subsequently heated in a microwave at 750 W for 20 minutes, with 5 minutes intervals. All the slides were then allowed to cool for 20 minutes, and then rinsed with phosphate buffered saline (PBS) wash buffer. The sections were quenched in 3% hydrogen peroxide (H_2_O_2_) containing 15 mmol/l sodium azide (NaN_3_) for 10 minutes to block endogenous peroxidase activity, followed with a rinse in PBS wash buffer. Ten microlitres of ready to use primary monoclonal mouse anti-human antibodies, each of p16INK4A, EGFR (clone EGFR-384-R-7-CE; Leica, Germany), pEGFR (clone EP774Y, Biocare Medical, USA) were applied, and incubated for 30 minutes at room temperature, and according to manufacturers’ instructions (Table 1). The sections were rinsed with wash buffer, followed with incubation in a visualization reagent for 30 minutes. This was followed with rinsing and incubation in a substrate-chromogen solution for 10 minutes to facilitate visualization. After the final rinse with PBS buffer, sections were counterstained with Mayer’s hematoxylin, dehydrated and mounted. For each run, normal skin, normal placenta and colon carcinoma specimens were used as positive controls for p16^INK4^, EGFR and pEGFR, respectively.

### DNA extraction from tissue blocks

DNA was extracted from 20 sections (5 μm) of the FFPE tissue blocks using digestion buffer, 50 mM Tris-pH 8.5, 1 mM EDTA, pH 8.0 and 0.5% Tween 20 (Sigma) and proteinase-K (Roche) at a final concentration of 500 μg/ml. The lysates were purified using EZ1 DNA Tissue Kit (Qiagen) and 1 μl of DNA were used in a reaction to verify DNA integrity, using beta globin and as described previously in other studies [56-59]. DNA extraction was successful in forty out of the fifty eight study cases, most probably due to poor tissue processing that resulted in DNA degradation.

### PCR testing for HPV and genotyping

For the detection of human papillomavirus (HPV) and the genotypes, PCR was performed, followed with DNA sequencing on ABI PRISM 310 Genetic Analyzer. The sequences compared with the GenBank database. In brief, the PCR reaction was performed in a final volume of 50 μl. Each PCR mixture contained 25 μl of AmpliTaq Gold PCR Master Mix 2X (Applied Biosystems, NJ USA), 30 pmol of primers (A1 5'- TTGGATCCATGTTAATWSAGCCWCCAAAATT –3' A2 5'- TTGGATCCTTATCAWATGCCCAYTGTACCAT –3'), 2 μl of MgCl_2_ (25 mM) 1 μl of either DNA or water. The cycling conditions were as follows: 10 minutes at 95°C activation step for Taq DNA polymerase, followed by 40 cycles each for 40 seconds at 95°C, 50 seconds at 55°C and 40 seconds at 72°C. The last cycle was followed by a final extension step of 10 minutes at 72°C. The PCR products were analyzed by electrophoresis on 2% agarose gel stained with ethidium bromide using the protocol described by Gleissner *et al*. [60].

Positive samples were typed using cut and purified DNA bands from agarose gel. The DNA were sequenced with an ABI PRISM 310 Genetic Analyzer (Applied Biosystems, Weiterstadt, Germany), with the Big Dye Terminator V1.1 cycle sequencing kit (Applied Biosystems, NJ, USA) using primer (A1). Sequences were then compared with the GenBank database as described by Sgueglia *et al.*[61].

### Statistical analysis

Data was then analysed for significant statistical co-relations using SPSS Version 16. One-tailed *p* < 0.05 at 95% CI was considered statistically significant.

## Competing interest

The authors declare that they have no competing interests.

## Authors’ contributions

Contributions: AM, JN, WO, MK, LP and NO, performed the experiments; ER and JN designed the overall study; WM, coordinated the work; RS, ER and LL contributed their expertise in the field of pathology; AM and JN wrote the work. All authors read and approved the final manuscript.
